# Engineering sigma factors and chaperones for enhanced heterologous lipoxygenase production in *Escherichia coli*

**DOI:** 10.1186/s13068-022-02206-x

**Published:** 2022-10-10

**Authors:** Cuiping Pang, Guoqiang Zhang, Song Liu, Jingwen Zhou, Jianghua Li, Guocheng Du

**Affiliations:** 1 National Engineering Research Center for Cereal Fermentation and Food Biomanufacturing, Jiangnan University, 1800 Lihu Road, Wuxi, 214122 Jiangsu China; 2 Science Center for Future Foods, Jiangnan University, Wuxi, 214122 China; 3 School of Biotechnology and Key Laboratory of Industrial Biotechnology, Ministry of Education, Jiangnan University, 1800 Lihu Road, Wuxi, 214122 Jiangsu China; 4 Engineering Research Center of Ministry of Education On Food Synthetic Biotechnology, Ministry of Education, Jiangnan University, 1800 Lihu Road, Wuxi, 214122 Jiangsu China

**Keywords:** Soluble expression, High-throughput screening, σ factor, Molecular chaperone

## Abstract

**Background:**

Lipoxygenase (EC. 1.13.11.12, LOX) can catalyze the addition of oxygen into polyunsaturated fatty acids to produce hydroperoxides, which are widely used in the food, chemical, and pharmaceutical industries. In recent years, the heterologous production of LOX by *Escherichia coli* has attracted extensive attention. However, overexpressed recombinant LOX in *E. coli* aggregates and forms insoluble inclusion bodies owing to protein misfolding.

**Results:**

In this study, a split green fluorescent protein-based screening method was developed to screen sigma (*σ*) factors and molecular chaperones for soluble LOX expression. Three mutant libraries of Skp, GroES, and RpoH was analyzed using the high-throughput screening method developed herein, and a series of mutants with significantly higher yield of soluble heterologous LOX were obtained. The soluble expression level of LOX in the isolated mutants increased by 4.2- to 5.3-fold. Further, the highest LOX activity (up to 6240 ± 269 U·g-DCW^−1^) was observed in *E. coli* REopt, with the regulatory factor mutants, RpoH and GroES. Based on RNA-Seq analysis of the selected strains, *E. coli* Eopt, *E. coli* Sopt, *E. coli* Ropt, and wild type, amino acid substitutions in σ factors and molecular chaperones regulated the expression level of genes related to gene replication, recombination, and repair. Furthermore, the regulatory factor mutants were identified to be beneficial to the soluble expression of two other heterologous proteins, amylase and bone morphological protein 12.

**Conclusion:**

In this study, a high-throughput screening method was developed for improved soluble LOX expression. The obtained positive mutants of the regulatory factor were analyzed and employed for the expression of other heterologous proteins, thus providing a potential solution for the inclusion-body protein.

**Supplementary Information:**

The online version contains supplementary material available at 10.1186/s13068-022-02206-x.

## Background

Lipoxygenases (EC. 1.13.11.12, LOX) are a family of non-heme iron oxidoreductases that catalyze the addition of oxygen to polyunsaturated fatty acids and are used in the food, chemical, pharmaceutical, and textile industries [1, 2]. At present, commercialized LOX is mainly derived from the extraction of plant tissues or fruits. As different batches of raw materials are employed, the quality of LOX is unstable. Owing to developments in enzyme engineering, the microbial production of LOX has increasingly demonstrated its advantages, including low cost and high purity.

*E. coli* is the most widely used host for the production of heterologous proteins, including LOX; however, overexpressed recombinant proteins always degrade or aggregate as insoluble and inactive inclusion bodies owing to misfolding [3]. Inclusion bodies have been demonstrated to be toxic to cells and affect their physiological functions [4]. Moreover, even if the expression level of the recombinant protein increases, optimizing the subsequent protein renaturation for inclusion bodies is time-consuming and will result in a low yield [5]. Therefore, promoting the soluble expression of recombinant proteins is an essential strategy for improving functional protein production. Currently, the strategies for increasing soluble protein yields mainly include decreasing the temperature of protein expression, optimizing expression conditions [6], optimizing expression hosts [7], and co-expressing through fusion with highly soluble protein tags (MBP, SUMO et al.) [8]. Another attractive alternate method for increasing soluble protein yields involves the engineering of or evolutionary pattern of the target protein itself or the regulatory protein to promote its soluble expression. For example, researchers have successfully used directed evolution to identify protein variants that are resistant to high temperatures [9], denaturants [10], or proteases [11].

Molecular chaperones and folding enzymes influence the folding of nascent protein and the refolding of misfolded polypeptides, thereby directly interfering protein aggregation, or directing misfolded or aggregated polypeptides to cellular clearance pathways [12]. The co-expression of chaperones can effectively promote the solubility of a target protein in vivo [13], and immobilized chaperones can improve the refolding efficiency of target proteins in vitro [14]. However, the extensive production of active recombinant protein via co-expression of chaperones is mainly dependent on the synergistic actions of chaperones in transcription, translation, folding, and post-translational modifications, which increases the difficulty of ensuring accurate predictions and formulating rational designs [15]. The combination of a target protein and chaperone is highly specific in most cases [16]. In fact, additional studies have shown that most chaperones are heat shock proteins that are mainly regulated by heat shock transcription factors [17]. Therefore, it is necessary to obtain and utilize transcription factors and chaperones to promote the soluble expression of target proteins.

Directed evolution could improve the efficient heterologous expression of heterologous proteins by increasing the stability and solubility of the protein in the host cell, but also requires substantial time and labor for improving target property of protein [12, 18]. Each round of traditional protein evolution is time-consuming, thereby rendering the prospect of developing improvements in soluble expression unattractive. A method that allows the rapid improvement of protein expression, coupled with the preservation or improvement of protein functions, would offer substantial benefits. Bimolecular fluorescence complementation technology (BiFC) has been recently applied to high-throughput screening and structural analysis of recombinant proteins [19]. BiFC relies on the interactions between the bait and prey proteins that combine two non-fluorescent split protein domains and subsequent co-folding into the β-barrel structure to form the chromophore [18]. For example, Zhang et al. [20] developed a novel fluorescent biosensor based on a split GFP for the screening of transpeptidase inhibitors.

Our previous study revealed numerous LOX inclusion bodies in the *E. coli* expression system [21]. To further facilitate the soluble expression of *Pseudomonas aeruginosa* LOX in *E. coli*, a split GFP-based soluble protein screening system was developed in this study, and used to promote heterologous LOX production with different regulatory factors and chaperones. The selected potential factors were characterized and applied to other heterologous proteins.

## Materials and methods

### Strains, plasmids and culture conditions

The *lox* gene derived from *Pseudomonas aeruginosa* [21], and the specific gene sequence is shown in Additional file 1: File S1. The strains, plasmids, and primers used in this work are listed in Additional file 1: Tables S1 and Additional file 1: Tables S2. *E. coli* JM109, used as the cloning host for plasmid construction, was growing in Luria–Bertani (LB) broth (5 g·L^−1^ yeast extract, 10 g·L^−1^ tryptone, and 10 g·L^−1^ NaCl), when necessary, with 1.5% Bacto agar. *E. coli* BL21 (DE3) was used as the expression strain in Terrific Broth medium (12 g·L^−1^ tryptone, 24 g·L^−1^ yeast extract, 5 g·L^−1^ glycerol, 2.31 g·L^−1^ KH_2_PO_4_, 16.84 g·L^−1^ K_2_HPO_4_) for fermentation. The ampicillin and chloramphenicol were added to the medium with 100 μg·ml^−1^ and 30 μg·ml^−1^ final concentration to maintain the plasmid if necessary.

### Construction of soluble protein screening system based on split GFP assembly

The vector pETDuet-1 has two expression cassettes designed for the co-expression of two target proteins. The recombinant plasmid pET Duet-1-GFP_1-10_ was obtained by inserting a GFP_1-10_ fragment into the first multiple cloning site (MCS) region. Another plasmid, pET Duet-1-GFP_11_, was constructed by inserting a GFP_11_ fragment into the second MCS region of pET Duet-1. Fragments of both GFP_1-10_ and GFP_11_ were inserted into the MCS regions of pET Duet-1 to construct the recombinant plasmid pET Duet-1-GFP_1-10_/GFP_11_. LOX was accordingly inserted into the 5′- and 3′-ends of the second expression frame GFP_11_ to obtain the recombinant plasmids pET Duet-1-GFP_1-10_/LOX-GFP_11_ and pET Duet-1-GFP_1-10_/GFP_11_-LOX. These plasmids were transformed into *E. coli* JM109 cells via heat shock transformation, and the positive clones obtained through colony PCR and DNA sequencing screening were transformed into *E. coli* BL21 (DE3) cells for subsequent protein expression experiments.

### Construction of *σ*-factor and molecular chaperone co-expression systems

Transcription factors used in protein transcription and molecular chaperones utilized in protein folding were selected for further experiments. Transcription factors included δ70 (*rpoD*), δ32 (*rpoH*), δ19 (*fecl*), and the β-subunit of the core enzyme (*rpoB*). Molecular chaperones included HSP40 (*DnaJ*), HSP60 (*GroEL*), HSP70 (*DnaK*), HSP90 (*HtpG*), HSP100 (*ClpA, ClpB*), sHSP (*ibpA, ibpB, skp, GroES, GrpE*) and the trigger factor TF. The pACYC Duet-1 vector was used to construct recombinant plasmids containing the corresponding transcription factors and molecular chaperones. To reduce metabolic stress, the promoter P_ssar_ was selected to be as promoter of these genes [22]. The above plasmids were screened for positive clones using heat shock transformation and colony PCR, and subsequently used for experiments after correct sequencing was performed.

### Construction of the GroES, Skp, RpoH mutant library

The plasmids, pACYC-GroES, pACYC-Skp, and pACYC-RpoH, were used as templates to design the error-prone PCR (epPCR) primer. The primer sequence is shown in Additional file 1: Table S2. The *GroES*, *skp*, *rpoH* genes, and vector fragment were amplified using forward/reverse primers from the corresponding plasmid. The following PCR cycle condition was employed: initial denaturation, 98 °C for 5 min; followed by 34 cycles of denaturation at 98 °C for 10 s; annealing at 55 °C for 5 s; and extension at 72 °C for 2–3.5 min. To remove the primary template, *Dpn*I was used to digest the PCR products. Numerous mutant sequences containing the target gene were obtained by random mutagenesis of the amplified fragments using the GeneMorph II Random Mutagenesis Kit (Agilent Technologies, California, America). The following amplification conditions was used for PCR: initial denaturation, 95 °C for 2 min; followed by 35 cycles of denaturation at 98 °C for 30 s; annealing at 55 °C for 30 s; and extension at 72 °C for 1 or 2 min. The isolated DNA mutant and vector fragments were assembled using the One Step Cloning Kit (Vazyme, Nanjing, China), yielding mutants plasmid library containing the target gene. The PCR products were purified on a SanPrep column (Sangon Biotech, Shanghai, China), and subsequently sequenced by Sangon Biotech Co. Ltd. (Shanghai, China). After recombination and transformation, the recombinant bacteria containing numerous mutant sequences were obtained. Thereafter, the colonies on the plate were washed and cultured in 1.5 ml LB medium for 1 h. After the plasmids were extracted and transformed into *E. coli* BL21 (DE3) containing pET Duet-1-GFP_1-10_/GFP_11_-LOX, the screening mutation library was constructed.

### High-throughput screening via split GFP

The mutant library was transformed into *E. coli* BL21 (DE3) containing pET Duet-1-GFP_1-10_/GFP_11_-LOX plasmid, and recovery was performed at 37 °C for 1 h. Antibiotics were subsequently added to prevent plasmid loss. When cells were cultured to an OD_600_ of 0.6–1, 0.1 mM IPTG was added to induce protein expression at 30 °C for 3 h. Subsequently, the cells were collected and washed three times with PBS buffer. Finally, the cells were resuspended in PBS buffer, and the OD_600_ was adjusted to 0.1. The remaining cells were stored in 15% glycerol at − 80 °C for future flow sorting. Cell sorting was performed using a Becton Dickenson FACSAria^™^ II Flow Cytometer (Becton, Dickinson and Company, New York, USA). The flow cytometry gating strategy included normally growing fluorescent cells. Two dual gates with forward and side scatter were used to remove more than one cell per droplet and ultimately sort the higher fluorescence intensity of ≤ 1% of positive colonies. Positive cells were sorted in LB medium and incubated for 1 h at 37 °C. Thereafter, the cells were coated on LB solid medium containing ampicillin and chloramphenicol.

After the cells sorted by flow cytometry developed colonies on the plates, the bacteria were picked and transferred to 96 shallow-well plates containing 200-μl LB media using a 10-μl sterilized gun tip, and incubated at 37 °C for 8–10 h. Thereafter, the colonies were transferred to 96 deep-well plates containing 800 μl of TB medium at 4% inoculum and incubated at 37 °C for 2 h. IPTG (0.1 mM) was added to induce LOX expression at 30 °C for 6 h. Four wells of each plated were reserved as controls (two positive and two negative controls). The culture was then collected by centrifugation and washed twice with 20 mM PBS (pH 7.4). The whole-cell fluorescence of these colonies was detected (excitation at 488 nm and emission at 520 nm) using a microplate spectrophotometer (BioTek, California, USA). Four rounds of sorting were performed prior to plasmid sequencing.

### Purification of LOX from the recombinant *E. coli*

LOX protein was purified by nickel column affinity chromatography. The specific process is as follows: the fermentation supernatant was collected by centrifugation at 4 °C 14,000 rpm for 30 min after cell lysis, then filtered used by 0.22-μm mixed cellulose membrane. Nickel affinity chromatography (His Trap TM FF crude, GE Healthcare) was performed by washing the beads with 5–10 column volumes of distilled water to eliminate the preservative and by equilibrating the column with 5–10 column volumes of binding buffer (20 mM phosphate buffer, PB buffer, 20 mM K_2_HPO_4_ and KH_2_PO_4_, pH 7.4). The sample was loaded into a nickel affinity chromatography column pre-equilibrated with buffer A (20 mM phosphate buffer, PB buffer, 20 mM K_2_HPO_4_ and KH_2_PO_4_, pH 7.4). After re-equilibration, the hetero protein was eluted with 15% buffer B (20 mM phosphate buffer, PB buffer, 20 mM K_2_HPO_4_ and KH_2_PO_4_, 500 mM imidazole, pH 7.4). The target LOX was eluted with 25% buffer B and then dialyzed overnight at 4 °C in the precooled buffer A to obtain pure protein. Since desalination is a sample dilution process, it is necessary to concentrate the desalinated LOX sample with a concentration tube for subsequent analysis.

### Assays for LOX activity

The catalytic activity of LOX was assayed according to the method of Pang [21]. One unit of LOX activity was defined as a 0.001 increase in absorbance value in one minute, at 470 nm. In all experiments, a “blank” was used to check for change in the absence of substrate, during the incubation period. Activities were measured in triplicate, and error bars indicate standard deviation from the mean.

### SDS-PAGE and protein concentration assay

Aliquots of *E. coli* lysate, supernatant, and pellets (re-suspended fraction) were mixed with SDS loading buffer (NuPAGE1 LDS Sample Buffer 4 ×, Fisher Scientific) at a ratio of 3:1 and sodium dodecyl sulfate–polyacrylamide gel electrophoresis (SDS-PAGE) was performed, as described previously [23]. Using bovine serum albumin as a standard, the protein concentration was determined by the Bradford method [24]. The relative quantitative method was used to evaluate the protein expression level. Protein bands were quantified by the Relative Quantity Tab in the Quantity tool of Image Lab software (Bio Rad, New York, USA).

### Transcriptional analyses

*E. coli* expressing LOX, GroES, Skp, RpoH, and the wild type, or one of the corresponding mutants, were grown in triplicate in TB media to the mid-log stage. RNA samples were extracted using a Bacteria Total RNA Isolation Kit (Sangon Biotech, Shanghai, China), according to the manufacturer’s protocol. RNA enrichment, library construction, sequencing, and bioinformatic analyses were performed by Sangon Biotech (Shanghai, China) using an Illumina high-throughput sequencing system. Raw RNASeq read counts were normalized for gene length and library size to calculate normalized reads per kilobase of transcript and transcripts per kilobase million (TPM). The TPM values were used as a normalized measurement of gene expression. This statistic combines DESeq size factor-normalized counts [25]. A differential gene expression analysis of each mutant compared with that of the wild-type samples was performed using DESeq. The samples relevant to each differential comparison were included in the analyses. Annotation classification was performed according to the KEGG public database, and enrichment analysis was conducted using the phyper function in the R software. The *p*-value was calculated, and FDR correction was applied to the *p*-value to obtain the *q*-value, where a *q*-value ≤ 0.05 was considered significantly enriched.

## Results and discussion

### Characterization of LOX soluble screening system based on split GFP

**Fig. 1:**
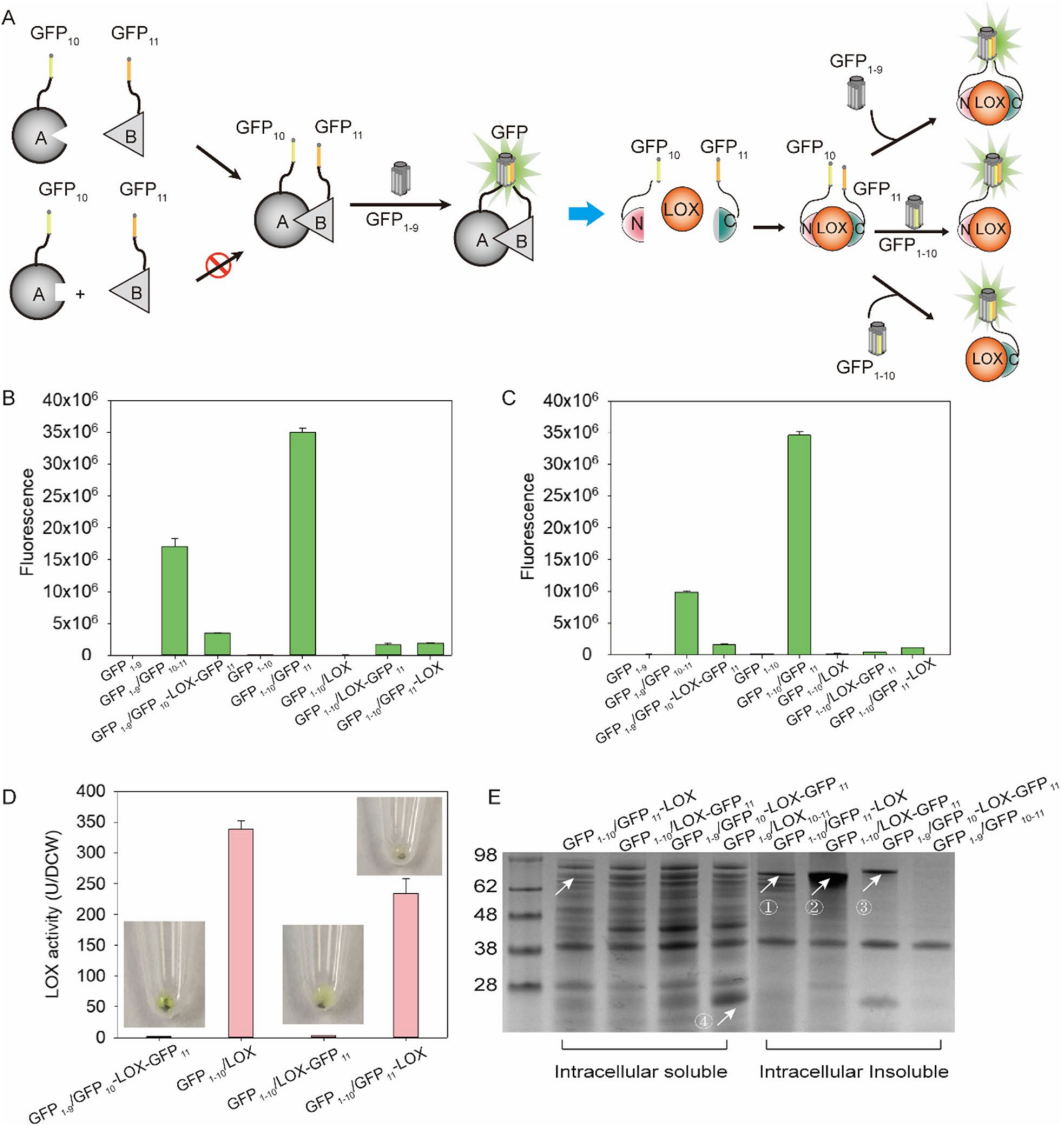
Construction of the screening system for soluble LOX expression. **A** Schematic diagram of the self-assembly protein–protein interaction of different domains in GFP (left). Soluble expression system with spontaneous assembly LOX fluorescence (right). **B** Fluorescence density analysis of the domain of split-GFP. **C** Fluorescence analysis of the soluble fraction of the self-assembly protein. **D** Enzyme activity and insoluble fraction fluorescence analysis using different screening systems. **E** Analysis of the expression level of LOX in different soluble expression systems

Self-associating bipartite/tripartite split GFP systems have been widely exploited for monitoring the soluble expression in vitro, localization, and trafficking of proteins [26]. Herein, a spontaneously assembling split GFP system was constructed, which comprised two short peptides, GFP_10_ (residues 194–212) and GFP_11_ (residues 213–233), and a large domain GFP_1–9_ (residues 1–193, detector) [26, 27]. This system involves the spontaneous assembly of two non-fluorescent split protein structural domains, the 1–9/1–10th β-strand of sfGFP (GFP_1–9_/GFP_1–10_) and the 10–11/11th β-strand (GFP_10–11_/GFP_11_), which subsequently co-fold into the β-barrel structure to form a chromophore (Fig. 1A). To establish a rapid screening system for soluble proteins, we constructed soluble protein expression systems for pET Duet-1-GFP_1–10_/GFP_11_ and pET Duet-1-GFP_1–9_/GFP_10–11_ using the self-assembly function of GFP_1–9_, GFP_10_, and GFP_11_. LOX was then employed as a target protein for soluble screening, which led to the construction of the plasmids, pET Duet-1-GFP_1–10_/LOX-GFP_11_, pET Duet-1-GFP_1–10_/GFP_11_-LOX, and pET Duet-1-GFP_1–9_/GFP_10_-LOX-GFP_11_ (Fig. 1A and Additional file 1: Fig. S1).

Different locations of LOX in the system may affect its solubility and folding, leading to the generation of a spontaneously assembled insoluble background signal [28]. As the catalytic active site of LOX is located at the C-terminal, LOX activity might be affected when the split GFP is fused at the C-terminal. Therefore, the availability of the screening system must be evaluated based on split GFP. All soluble protein screening systems in the entire cell showed fluorescence; however, the intensity of the fluorescence was far below that of the system without fused LOX (Fig. 1B). Furthermore, the intracellular supernatant extracted by sonication displayed a similar fluorescence change trend (Fig. 1C). The enzyme activity revealed that only pET Duet-1-GFP_1–10_/GFP_11_-LOX had discernible activity (Fig. 1D). Notably, other combinations without activity may be affected by the fusion of GFP_11_ at the C-terminal of LOX. Cell precipitation with pET duet-1-GFP_1–9_/GFP_10_-LOX-GFP_11_ and pET duet-1-GFP_1–10_/LOX-GFP_11_ resulted in visible fluorescence after centrifugation, whereas cells with pET duet-1-GFP_1–10_/LOX-GFP_11_ could not be detected (Fig. 1D). These findings indicated that the fluorescence of the insoluble fragments in the cell with pET Duet-1-GFP_1–10_/GFP_11_-LOX was lower than that in the entire cell, and the background interference of this strain was minimal. In contrast, the fusion of GFP_11_ at the C-terminal of LOX affected the activity and increased the content of the inclusion body (Fig. 1D). SDS-PAGE revealed the content of soluble fusion protein and inclusion bodies in the above three systems. The inclusion body content of cells with pET duet-1-GFP_1–10_/LOX-GFP_11_ was the highest and that of pET Duet-1-GFP_1–10_/GFP_11_-LOX was the lowest (Fig. 1E), showing that this system has slight influence on LOX expression.

To further determine whether the screening system can effectively reflect soluble protein expression levels, the relationship between the soluble protein concentration, fluorescence density, and enzyme activity was investigated. The fluorescence intensity was found to increase as the protein concentration varied from 0.2 mg·ml^−1^ to 2.0 mg·ml^−1^ (Additional file 1: Fig. S2A). Further, the change in enzymatic activity was found to range from 85 to 2200 U·g-DCW^−1^, thereby indicating a good linear relationship between the fluorescence density and soluble protein level (Additional file 1: Fig. S2B). Overall, pET Duet-1-GFP_1–10_/GFP_11_-LOX could be used for high-throughput screening of soluble LOX expression.

### Screening of molecular chaperones and σ factors for soluble LOX expression

**Fig. 2:**
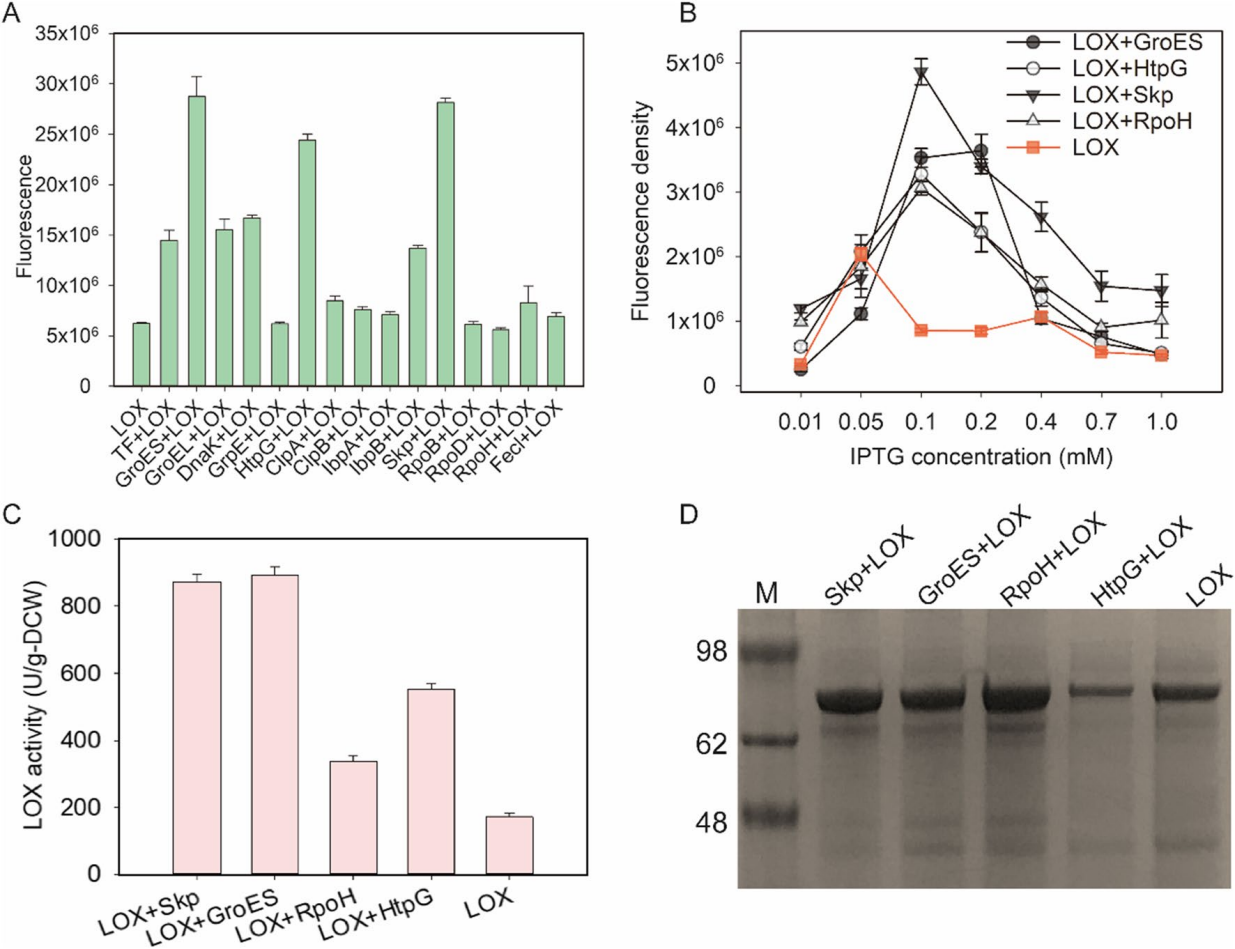
Screening and characterization of the soluble expression factors. **A** Molecular chaperone and transcription factor screening. **B** Effect of IPTG concentration on LOX soluble expression. **C** Effect of optimized induction concentration on LOX activity. **D** Effect of optimized induction concentration on LOX expression level

In our previous study, LOX was expressed in *E. coli*, and its inclusion bodies were slightly improved using a combination of strategies [21]. In this study, the effects of molecular chaperones HSP60 (*GroEL*), HSP70 (*DnaK*, *DnaJ*, and *GrpE*), HSP90 (*HtpG*), HSP100 (*ClpA*, *ClpB*), sHSP (*ibpA*, *ibpB*, *GroES*, *skp*)) and σ factors (*rpoB*, *rpoC*, *rpoD*, *rpoH*, and *fecl*) on the soluble expression of LOX were compared. These factors were initially screened using the developed split GFP system. Most molecular chaperones could significantly increase the fluorescence intensity and promote the soluble expression of LOX, especially GroES, Skp, and HtpG, when 1 mM IPTG was added as inducer. Compared with the wild-type strain, the fluorescence intensity increased by 4.6-, 4.5-, and 3.9-times after 12 h of culture owing to co-expression with GroES, Skp, and HtpG, respectively (Fig. 2A). In addition, the overexpression of RpoH affected the soluble LOX expression at 30 °C (Fig. 2A). This finding is due to the expression of more than 30 heat shock genes being influenced by the σ factor, RpoH, in *E. coli* [29]. Therefore, the molecular chaperones, GroES, HtpG, and Skp, and the transcription factor, RpoH, were selected for a further study.

Considering the metabolic pressure of the induced expression for cells, the IPTG concentration for co-expression of LOX with Skp, GroES, HtpG, and RpoH was optimized. To shorten the screening time, the culture time was set as 6 h in the follow-up experiment. Based on the fluorescence density results, when the IPTG concentration for LOX expression was reduced from 0.4 to 0.1 mM, the co-expression of GroES, Skp, HtpG, and RpoH increased the soluble expression level by 3.3-, 4.6-, 3.1-, and 2.9-fold (Fig. 2B). The LOX enzyme activity was also increased by 97% to 420% in the different mutants (Fig. 2C). When the concentration of IPTG was 0.1 mM, the overall expression levels of LOX (including soluble and insoluble LOX) were improved by the co-expression of Skp, GroES, and RpoH, but was reduced by the co-expression of HtpG (Fig. 2D). Therefore, Skp, GroES, and RpoH were selected for directed evolution to further improve soluble LOX expression levels. Mo et al. increased soluble expression levels and enzyme activity through directed evolution of the thermal esterase, Aaeo1, based on split GFP [30]. However, the soluble expression of recombinant proteins is negatively correlated with their activity. Accordingly, more time and labor cost were required to obtain the target mutant [31].

### Directed evolution of GroES, Skp, and RpoH for soluble LOX expression

**Fig. 3:**
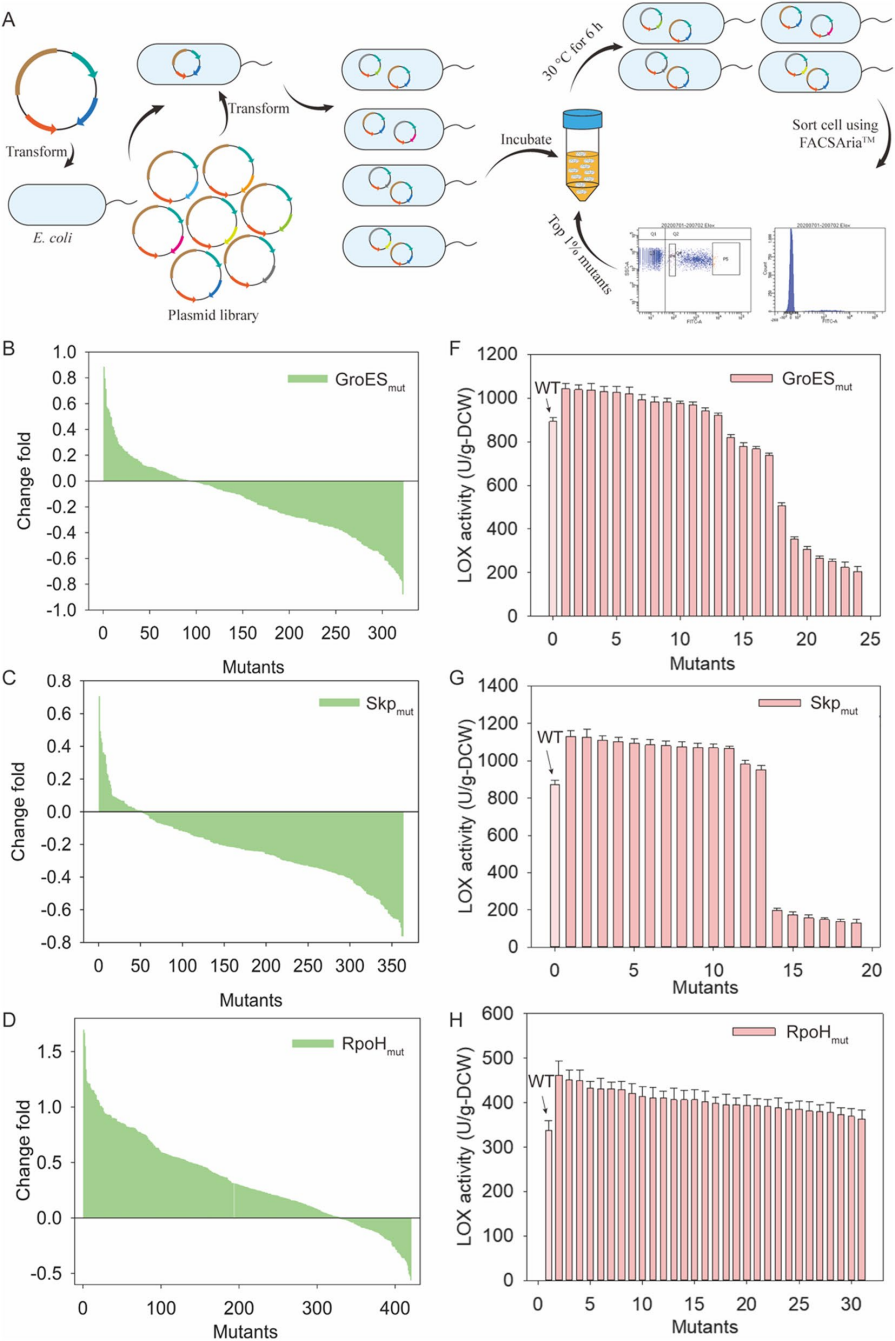
Directed evolution of the molecular chaperones and σ factor. **A** Flow screening process diagram. **B**–**D** Effect of the GroES, Skp, and RpoH mutants on the level of soluble LOX. **E**–**G** Effect of the GroES, Skp, and RpoH mutants on LOX activity

Mutant libraries of GroES, Skp, and RpoH were constructed using error-prone PCR. At the initial stage of library construction, 24 single colonies were randomly selected and tested to derive the mutation rates. Further sequencing revealed that the mutation rate was 85%, with 1–3 mutation sites per kb. Based on the developed screening system for soluble expression, the mutants with enhanced expression levels were sorted using fluorescence-activated cell sorting (FACS) (Fig. 3A).

The biosensor used in the FACS screening method had two main limitations. First, the plasmid was unstable when the temperature of expressed LOX was above 30 °C, resulting in a decrease in the level of LOX expression. Second, the differing growth statuses of cells occasionally led to increased protein contents in large cells, further leading to a stronger fluorescence signal. To maintain the stability of LOX expression, the plasmid stability region, parABS, was inserted at the *Sap*I digestion site of the pET Duet-1-GFP_1–10_/GFP_11_-LOX soluble screening system to ensure plasmid stability. The relative fluorescence intensity (FI/OD_600_) was used as a screening standard, and the sorted cells were further evaluated in 96-well plates. The positive rates of GroES, Skp, and RpoH were 29%, 14%, and 78%, respectively (Fig. 3B–D). Further, the optimal soluble expression levels of LOX with the mutants, GroES, Skp, and RpoH, had increased by 88%, 70%, and 169%, respectively (Fig. 3B–D). The mutants with varying fluorescence intensities were randomly selected for enzyme activity detection. The change in LOX activity and fluorescence intensity was consistent (Fig. 3E–G), which indicates that the directed evolution of *σ* factors and molecular chaperones is feasible using the soluble protein screening system.

**Fig. 4:**
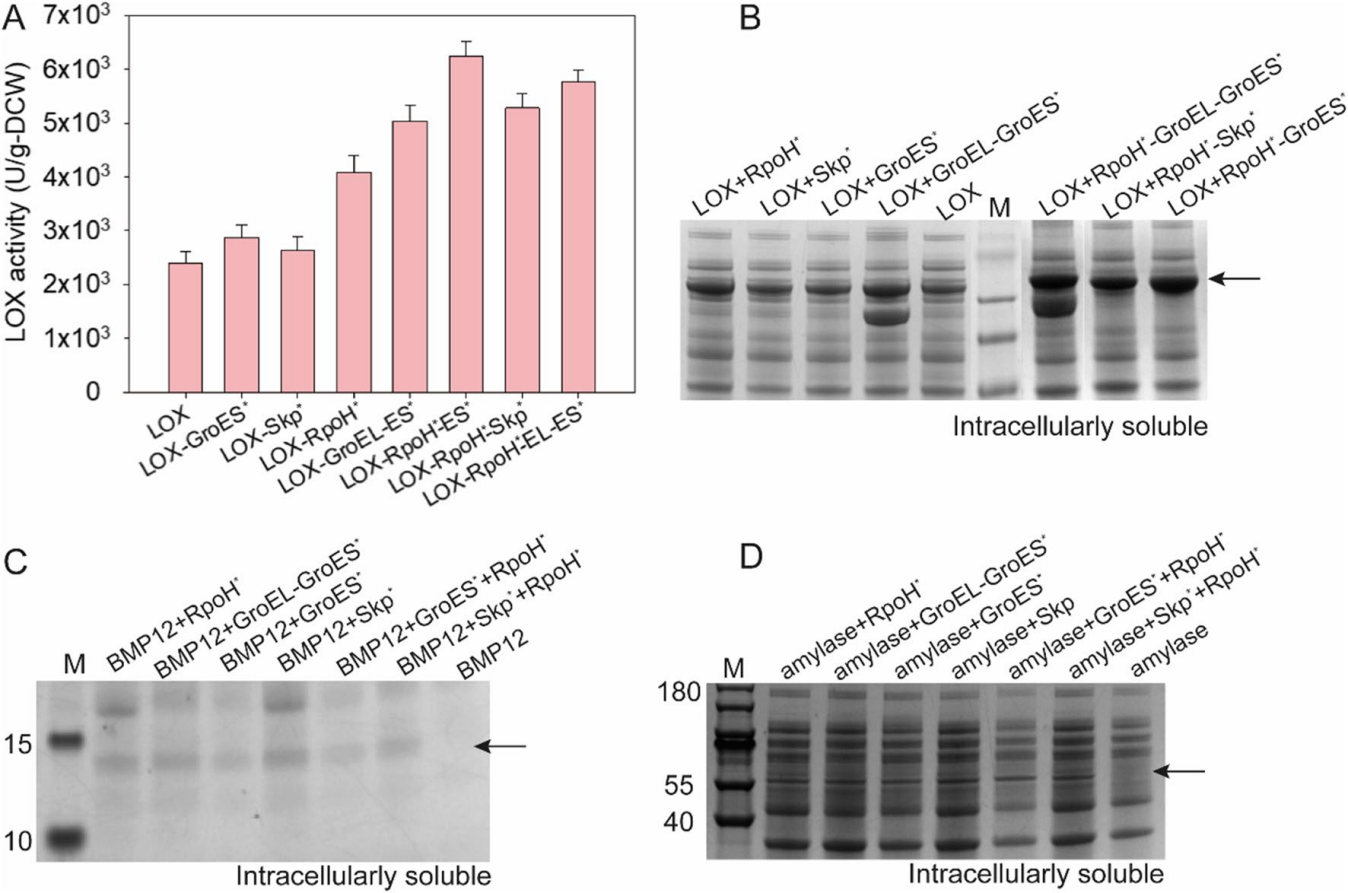
Effects of different positive mutants on the soluble expression of two other heterologous proteins. **A** Effects of different positive mutants on LOX activity. **B** Effects of different positive mutants on LOX soluble expression level. **C** Effects of mutants on the soluble expression of BMP12. **D** Effects of mutants on the soluble expression of α-amylase. *represents the optimized mutant

GroEL and GroES were previously reported to be essential molecular chaperones at all growth stages of *E. coli* [12]. GroES can effectively assist in folding and release of peptide chains in the cavity of the GroEL protein. Misfolding and aggregation of proteins can also be relieved by the chaperone, Skp, when transported between the inner and outer membranes. The RpoH regulates the synthesis of heat shock proteins at the transcriptional level. To enhance the soluble expression level of LOX, the combinations of different *σ* factors and chaperones were characterized. The highest LOX activity of 6240 ± 269 U·g-DCW^−1^ was achieved at 30 °C (Fig. 4A). When RpoH_mut_ was co-expressed with the GroEL-GroES_mut_ and Skp_mut_ mutants in *E. coli* REopt, the soluble LOX content increased by 1.9-fold (Fig. 4B, Additional file 1: Table S3). Our results indicate that *σ* factors and chaperones could synergistically regulate the expression level of LOX. In our study, the molecular chaperones and *σ* factors promoted soluble LOX expression and improved the overall expression level of the target protein. However, the limited intracellular space led to the aggregation of numerous proteins. As a result, this strategy did not completely solve the inclusion body limitation of LOX.

To test the practicability of the selected molecular chaperones and *σ* factor, two other proteins, namely α-amylase and bone morphological protein 12 (BMP12), were evaluated to derive their soluble expression. Notably, the obtained molecular chaperones and *σ* factors could effectively promote soluble expression of α-amylase and BMP12 (Fig. 4C, D).

### Sequence analysis of the GroES, Skp, and RpoH mutations

GroES is a 10-kDa dome-shaped seven-membered ring subunit that binds to the end of the GroEL cylinder to form a cage wrapped in a folded substrate protein. Each GroES subunit contains a flexible ring sequence [32]. During the evolution of GroES, mutants with improved soluble LOX expression were generated. Among them, numerous mutations were mainly concentrated in the first 24 amino acids of the N-terminal or the first 34 amino acids of the C-terminal (Additional file 1: Table. S3). These mutations may increase the flexibility of the mobile loop, which helps LOX to fold more effectively into the GroEL bucket. Previous studies have shown that the mutant V26A/Y71H/V83M of GroES is favorable for GFP folding [33]. In our study, we also found that the sites (G24, E82) near V26 and V83 can also promote protein folding. In addition, GroES binding causes significant conformational change in GroEL, approximately doubling the size of the central cavity and creating a relatively polar environment wherein the substrate folds [34]. Changes in GroEL/ES expression levels can also alter RpoH activity [35]. GroES mutants may increase other molecular chaperone levels through heat shock response while partially enhancing LOX folding.

The Skp molecular chaperone is a functional homologous trimer with a jellyfish-like structure. This structure can protect outer membrane proteins from misfolding and aggregation when transported between the inner and outer membranes [36–38]. In the Skp evolution process, the mutations that promoted the soluble expression of LOX were mainly located between 59–110 amino acids (Additional file 1: Table S3). Previous studies have shown that the Skp hydrophobic cavity can hold a folded protein that is approximately 25 kDa [39]. However, in this study, LOX was a 66-kDa protein. Therefore, these mutations may effectively increase the adaptability of the hydrophobic cavity to the LOX structure to promote the expression of soluble LOX.

RpoH, a *σ* factor with a molecular weight of 32 kDa, strongly influences the heat-shock response of *E. coli*, and can effectively regulate the action rates of molecular chaperones (DnaK, DnaJ, GroEL, GroES, GrpE, etc.) and proteolytic enzymes [40]. In previous studies, RpoH-83 and -84 had the highest conservatism, and their mutations rendered RpoH inactive [29]. During the directed evolution of RpoH, the mutation sites of beneficial mutants are scattered and have no obvious rules (Additional file 1: Table S3). Studies have shown that RpoH is more conservative, and its dynamic fine-tuning may seriously affect growth obstacles under high-temperature conditions [41]. The beneficial mutation of RpoH in our study effectively promoted the soluble expression of LOX and two other heterologous proteins.

### Transcriptome analyses of the selected mutants

**Fig. 5:**
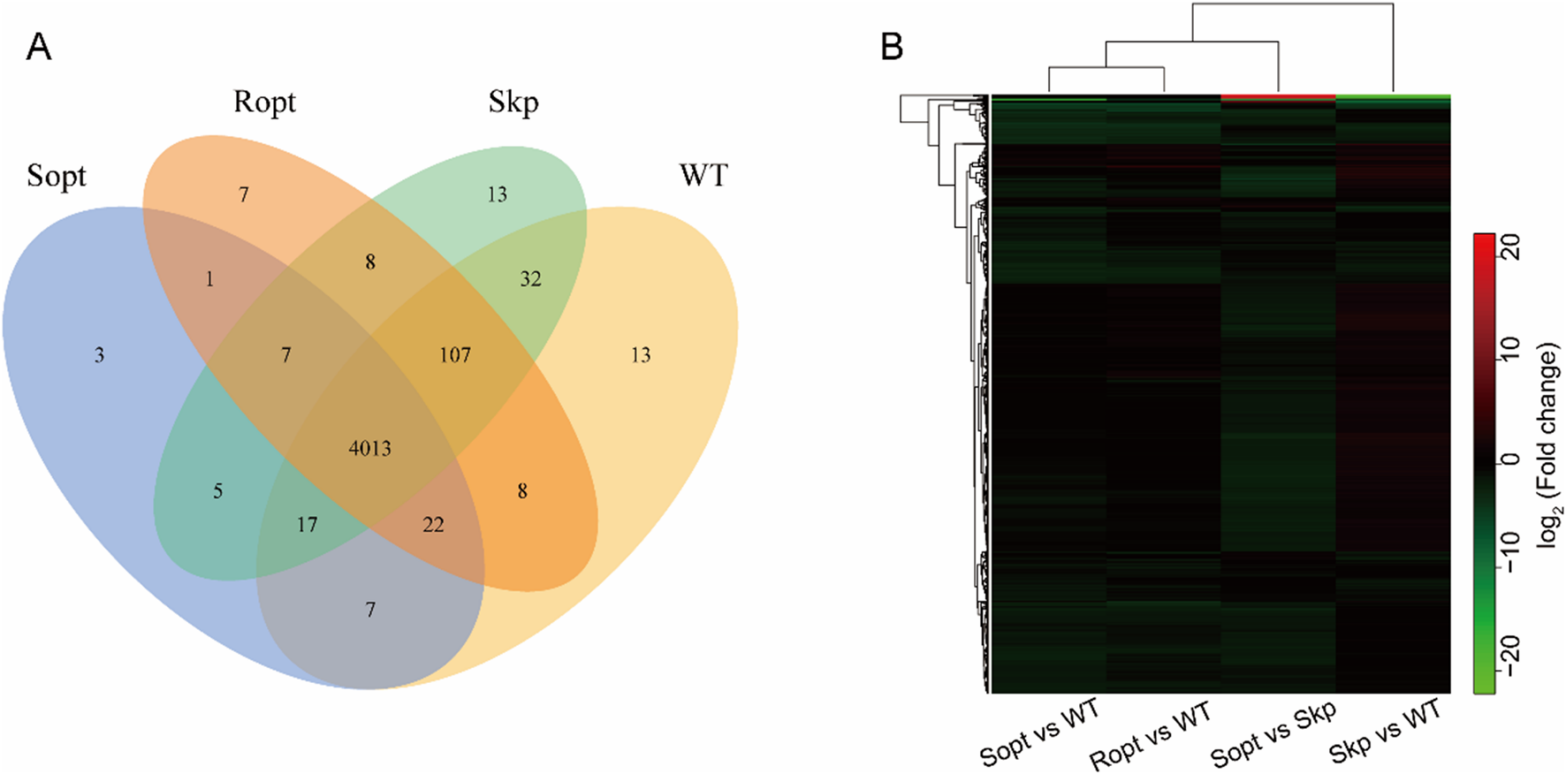
Effects of the positive mutant of Skp, RpoH, on LOX soluble expression in *E. coli*. **A** Three-way Venn diagram showing the number of co-expressed genes (adjusted *p*value ≤ 0.05) between *E. coli* expressing LOX, E. coli co-expressing LOX with the wild-type Skp, and E. coli co-expressing LOX with mutants of Skp and RpoH. **B** Heatmap showing differentially expressed genes (fold change 3, adjusted *p*-value ≤ 0.05) between *E. coli* expressing LOX, E. coli co-expressing LOX with the wild-type Skp, and *E. coli* co-expressing LOX with mutants of Skp and RpoH. Rows (genes) are clustered hierarchically

To investigate the effect of GroES, Skp, and RpoH on the soluble expression level of LOX, the transcriptomes of optimal strains with the GroES, Skp, and RpoH optimal mutants or the wild type were sequenced and analyzed to derive the possible regulation mechanism of LOX soluble expression based on differences in the gene expression of the mutants. The transcriptome data revealed 4013 expression genes in all strains (Fig. 5A), 16 of which were significantly differentially expressed (Additional file 1: Fig. S3A). Compared to the wild-type strain, strain Sopt, which had Skp mutant overexpression, displayed significant changes in its global transcription profiles, with 946 upregulated genes and 378 downregulated genes (Additional file 1: Fig. S3B). Thus, the introduction of genes for the overexpression of Skp can have a high impact on the global regulation of protein synthesis in the host. Of note, the transcriptional patterns of strains Sopt displayed considerable differences from those of the wild-type strain, with 35 upregulated genes and 736 downregulated genes (Additional file 1: Fig. S3B). Interestingly, the transcriptional patterns of strains Sopt showed similar significant differences relative to those of strain Sopt, with 42 upregulated genes and 679 downregulated genes (Additional file 1: Fig. S3B). These findings clearly demonstrate that the global regulation of protein synthesis in the host underwent remarkable changes after directed evolution of the molecular chaperone and *σ* factor. Further, these results highlight the significant value of molecular chaperone and *σ* factor evolution for the optimization of cellular protein synthesis. Several up/downregulated genes were observed among cells expressing different GroES, Skp, and RpoH mutants based on hierarchical cluster analysis of differential genes (Fig. 5B).

As the soluble expression capacity of LOX was used as a screening marker, the changes in the metabolism and regulation of these mutant strains should be conducive to the production of soluble LOX. To investigate this hypothesis, Clusters of Orthologous Groups of proteins (COG) and Kyoto encyclopedia of genes and genomes (KEGG) pathway enrichment analyses were performed to identify global regulation differences between the evolved strains and the chassis strain. Among them, changes in genes related to cellular processes, metabolism, organismal systems, environmental information, and genetic information processing were highlighted among the evolved strains (Additional files 1: Figs. S4, S5). Metabolic differences were observed among cells expressing different types of mutants based on KEGG pathway enrichment analysis. Changes in ribosomal, homologous recombination, and the carbon and nitrogen metabolism pathways were highlighted among the metabolic variations in the mutant strains (Additional file 1: Fig. S5A–C). Significant changes were suggested to occur in the global protein synthesis networks of these mutant strains. Compared with the control strains, the genes with significant differences in their expression level were mainly involved in DNA replication (*rnhB*, *dnaE*, and *dnaA*), homologous recombination (*dnaN*), mismatch repair (*mfd*), folding sorting (*ropE*), and maintenance of ribosomal homeostasis (*rsmG*, *mgtL*) in the mutant strains (Additional file 1: Fig. S5D). The expression level change of these genes could coordinate the balance between diverse cellular protein synthesis events and LOX production. Further, the changes in these pathways could enhance the efficiency of the initiation and elongation stages of DNA replication [42], reduce mismatch repair probability [43, 44], and increase the stability of ribosomes [45] and the accuracy of protein translation [46]. Therefore, the directed evolution of molecular chaperones and σ factor confirmed that they could regulate cell metabolism and protein function balance from the global protein synthesis level and promote the soluble expression of LOX.

## Conclusion

In this study, a split GFP-based screening system was developed to detect soluble LOX. Subsequently, transcription regulation factors and chaperone mutant libraries were constructed and screened to promote the soluble expression of LOX. The optimal mutants of GroES, Skp, and RpoH increased the soluble expression level of LOX by 4.2-, 5.3-, and 4.6-fold, respectively. Further, the activity of LOX was 6240 ± 269 U·g-DCW^−1^. RNA-Seq analyses suggested that amino acid substitutions in GroES, Skp, and RpoH altered the expression levels of genes related to gene replication, transcription, recombination, and repair. Consequently, fine-tuning of the transcriptional translation regulatory networks increased LOX activity by up to 420%. The obtained positive mutants of the regulatory factor were also applied for the expression of other heterologous proteins, namely α-amylase and BMP12, which provided a potential solution for inclusion-body protein. These results provide a potential strategy for cell factories construction and lay the foundation for further strain engineering towards boosting heterologous protein production.

## Supplementary Information

**Additional file 1: Fig S1.** Schematic diagram of spontaneous assembly fluorescent plasmid. **Fig S2.** Establishment of the relationship between LOX activity, fluorescence intensity, LOX soluble level. **A**. Correlation between fluorescence intensity and protein concentration; **B**. Correlation between fluorescence intensity and LOX activity. **Fig S3.** Clusters of Orthologous Groups of proteins (COG) **A **and Kyoto Encyclopedia of Genes and Genomes (KEGG) **B** analysis of common differential gene **A** Three-way Venn diagram showing the number of genes differentially expressed (fold change 3, adjusted *p*value≤0.05) between *E. coli* expressing LOX, *E. coli* co-expressing LOX with the wild-type Skp and *E. coli* co-expressing LOX with mutants of Skp and RpoH. **B** Genes differently expressed analysis statistical histogram. The horizontal axis is the name of the difference comparison, and the vertical axis is the number of up-down difference genes. **Fig S4.** Significant enrichment scatter of Orthologous Groups of proteins (COG) analysis of common differential gene. **A** Significant enrichment COG scatter diagram of differentially downregulated genes between S and WT. **B** Significant enrichment COG scatter diagram of differentially upregulated genes between S and WT. **C** Significant enrichment COG scatter diagram of differentially downregulated genes between Sopt and WT. **D** Significant enrichment COG scatter diagram of differentially upregulated genes between Sopt and WT. **E** Significant enrichment COG scatter diagram of differentially downregulated genes between Ropt and WT. **F** Significant enrichment COG scatter diagram of differentially upregulated genes between S and WT. The vertical axis represents the function annotation information, and the horizontal axis represents the rich factor corresponding to the function. The size of Qvalue is represented by the color of the dot. The smaller the Qvalue, the closer the color is to red. The dot size represents the number of differential genes contained in each function. **Fig. S5** Kyoto Encyclopedia of Genes and Genomes (KEGG) analysis of common differential gene. **A**–**C** Scatter diagram of significant enrichment function of differently expressed genes in the metabolic pathways between Sopt and WT, Sopt and S, Ropt and WT, respectively. The vertical axis represents the function annotation information, and the horizontal axis represents the rich factor corresponding to the function. The size of Qvalue is represented by the color of the dot. The smaller the *q*value, the closer the color is to red. The dot size represents the number of differential genes contained in each function. **D** Heatmap showing differentially expressed genes (fold change 3, adjusted *p*-value≤0.05) between *E. coli* expressing LOX, *E. coli* co-expressing LOX with the wild-type skp and *E. coli* co-expressing LOX with mutants of Skp. Rows (genes) were clustered hierarchically. **Table S1.** Plasmids and strains used in this work. **Table S2.** Oligonucleotides used in this study. **Table S3.** Mutant residue analysis of GroES, Skp and RpoH directed evolution Analysis of LOX expression level by Image Lab.
