# Supplementary material for: Engineering sigma factors and chaperones for enhanced heterologous lipoxygenase production in Escherichia coli
Source: Biotechnol Biofuels Bioprod. 2022 Oct 10;15:105. doi: 10.1186/s13068-022-02206-x (PMC9552429; doi:10.1186/s13068-022-02206-x)
Supplement: Supplementary file 1 — Additional file 1: Fig S1. Schematic diagram of spontaneous assembly fluorescent plasmid. Fig S2. Establishment of the relationship between LOX activity, fluorescence intensity, LOX soluble level. A. Correlation between fluorescence intensity and protein concentration; B. Correlation between fluorescence intensity and LOX activity. Fig S3. Clusters of Orthologous Groups of proteins (COG) A and Kyoto Encyclopedia of Genes and Genomes (KEGG) B analysis of common differential gene A Three-way Venn diagram showing the number of genes differentially expressed (fold change 3, adjusted pvalue≤0.05) between E. coli expressing LOX, E. coli co-expressing LOX with the wild-type Skp and E. coli co-expressing LOX with mutants of Skp and RpoH. B Genes differently expressed analysis statistical histogram. The horizontal axis is the name of the difference comparison, and the vertical axis is the number of up-down difference genes. Fig S4. Significant enrichment scatter of Orthologous Groups of proteins (COG) analysis of common differential gene. A Significant enrichment COG scatter diagram of differentially downregulated genes between S and WT. B Significant enrichment COG scatter diagram of differentially upregulated genes between S and WT. C Significant enrichment COG scatter diagram of differentially downregulated genes between Sopt and WT. D Significant enrichment COG scatter diagram of differentially upregulated genes between Sopt and WT. E Significant enrichment COG scatter diagram of differentially downregulated genes between Ropt and WT. F Significant enrichment COG scatter diagram of differentially upregulated genes between S and WT. The vertical axis represents the function annotation information, and the horizontal axis represents the rich factor corresponding to the function. The size of Qvalue is represented by the color of the dot. The smaller the Qvalue, the closer the color is to red. The dot size represents the number of differential genes contained in each functio [file 13068_2022_2206_MOESM1_ESM.docx]

**Supporting Information**

**Engineering sigma factors and chaperones for enhanced heterologous lipoxygenase production in *Escherichia coli***

Cuiping Pang^1,2^, Guoqiang Zhang^1,2,4^*, Song Liu^1,2,4^, Jingwen Zhou^1,2,4^, Jianghua Li^2,3,4^, Guocheng Du^2,3,4^

^1^National Engineering Research Center for Cereal Fermentation and Food Biomanufacturing, Jiangnan University, 1800 Lihu Road, Wuxi, Jiangsu 214122, China.

^2^Science Center for Future Foods, Jiangnan University, Wuxi, 214122, China.

^3^School of Biotechnology and Key Laboratory of Industrial Biotechnology, Ministry of Education, Jiangnan University, 1800 Lihu Road, Wuxi, Jiangsu 214122, China.

^4^Engineering Research Center of Ministry of Education on Food Synthetic Biotechnology, Ministry of Education, Jiangnan University, 1800 Lihu Road, Wuxi, Jiangsu 214122, China.

*Corresponding authors: Guoqiang Zhang

Mailing address: National Engineering Research Center for Cereal Fermentation and Food Biomanufacturing, Jiangnan University, 1800 Lihu Road, Wuxi, Jiangsu 214122, China

Phone: +86-510-85911286, Fax: +86-510-85911286

E-mail: [gqzhang@jiangnan.edu.cn](mailto:gqzhang@jiangnan.edu.cn); gqzhang2008@163.com


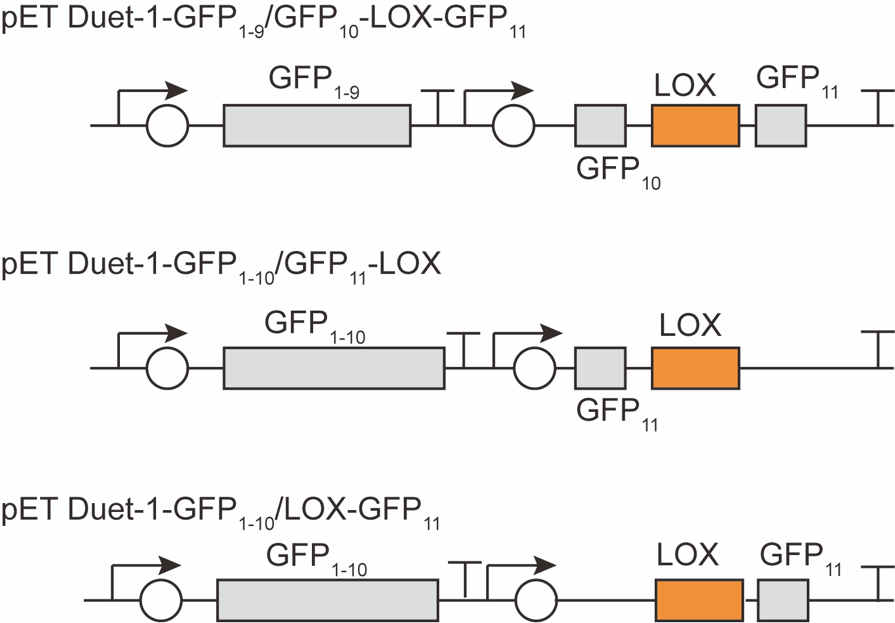


**Fig. S1 Schematic diagram of spontaneous assembly fluorescent plasmid**


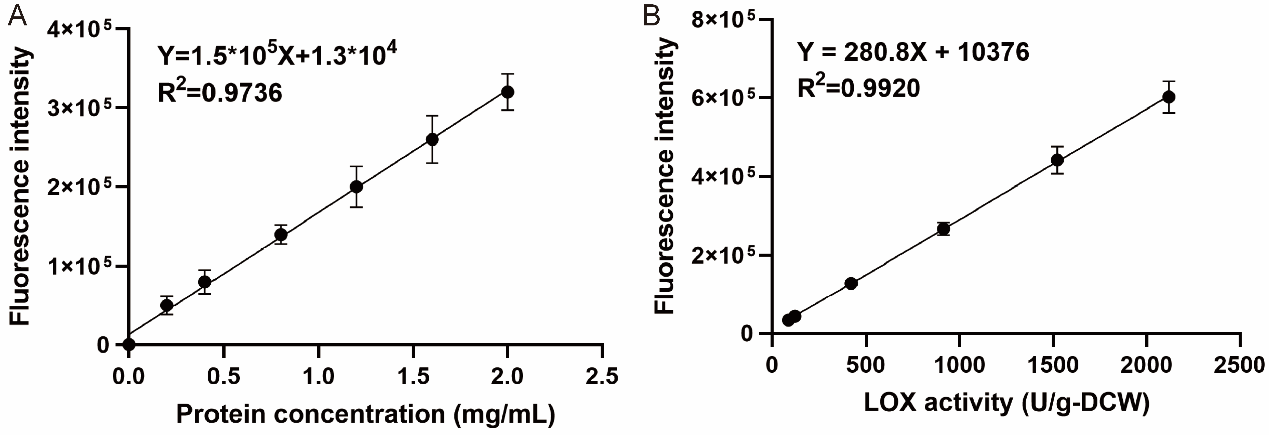


**Fig. S2 Establishment of the relationship between LOX activity, fluorescence intensity, LOX soluble level.** A. Correlation between fluorescence intensity and protein concentration; B. Correlation between fluorescence intensity and LOX activity.


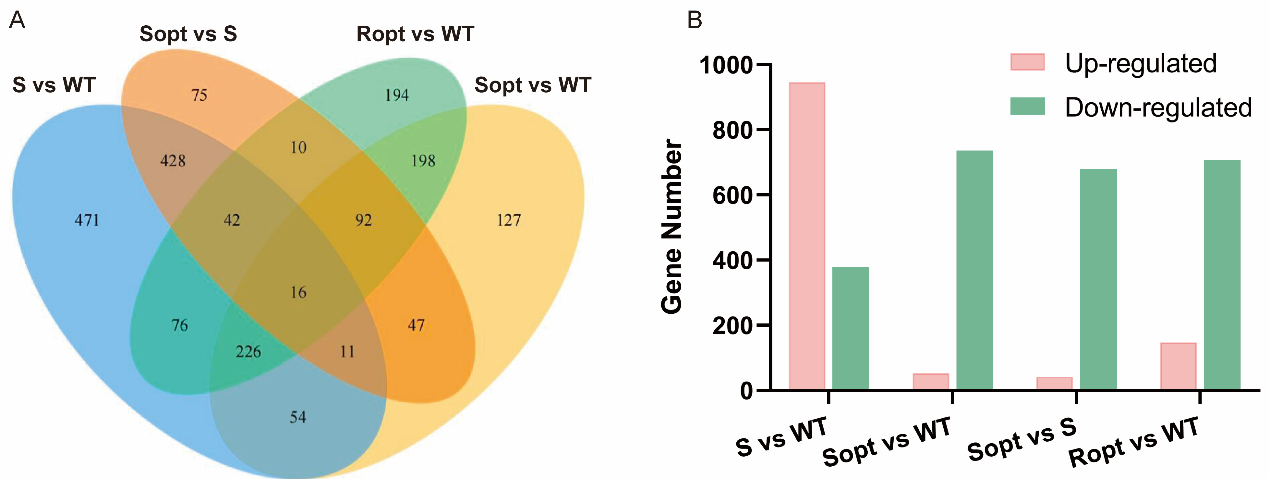


**Fig. S3 Clusters of Orthologous Groups of proteins (COG) (A) and Kyoto Encyclopedia of Genes and Genomes (KEGG) (B) analysis of common differential gene** (A) Three-way Venn diagram showing the number of genes differentially expressed (fold change 3, adjusted pvalue≤0.05) between *E. coli* expressing LOX, *E. coli* co-expressing LOX with the wild-type Skp and *E. coli* co-expressing LOX with mutants of Skp and RpoH. (B) Genes differently expressed analysis statistical histogram. The horizontal axis is the name of the difference comparison, and the vertical axis is the number of up-down difference genes.


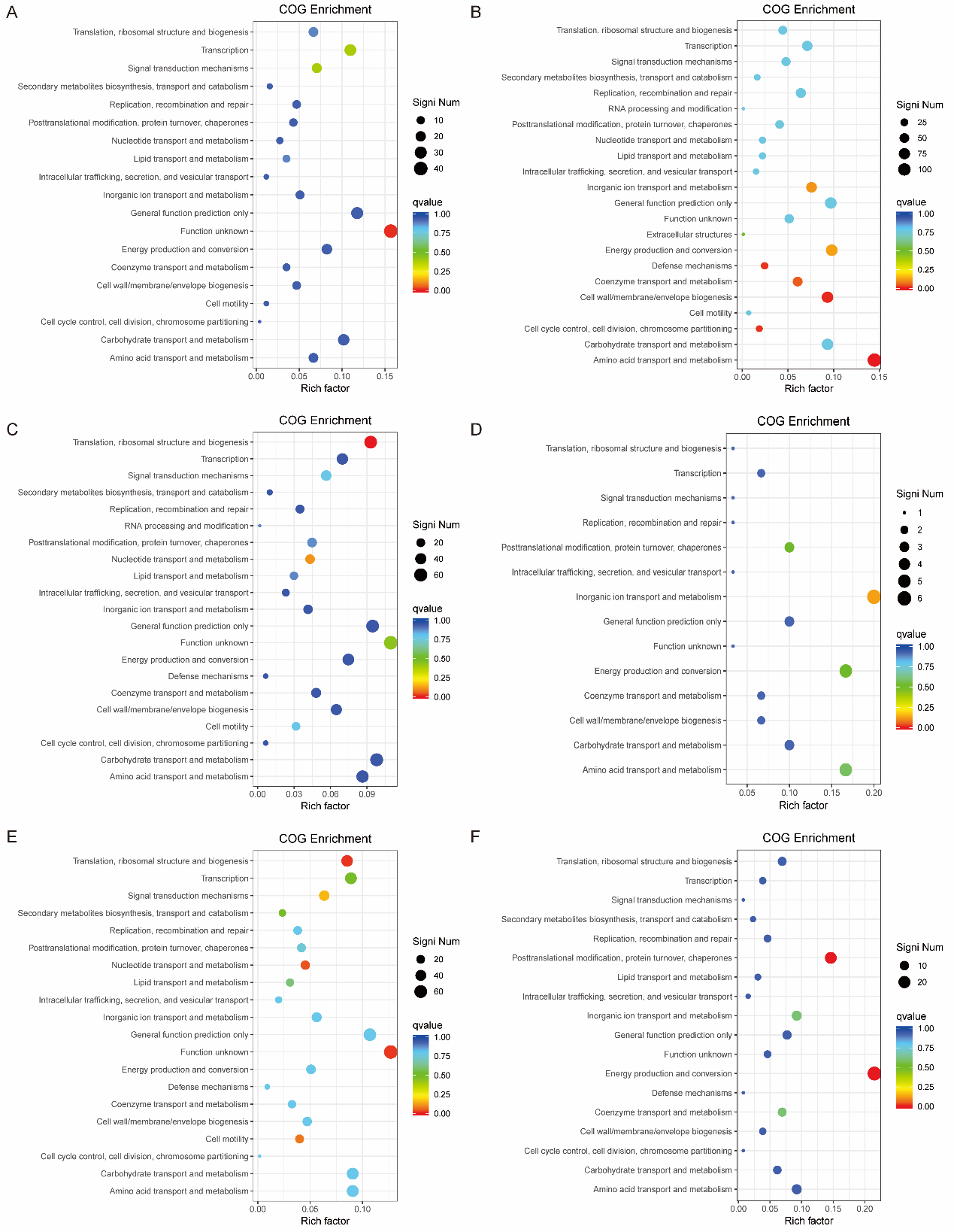


**Fig. S4** Significant enrichment **s**catter **of Orthologous Groups of proteins (COG) analysis of common differential gene.** (A) Significant enrichment COG scatter diagram of differentially downregulated genes between S and WT. **(**B) Significant enrichment COG scatter diagram of differentially upregulated genes between S and WT. **(**C) Significant enrichment COG scatter diagram of differentially downregulated genes between Sopt and WT. (D) Significant enrichment COG scatter diagram of differentially upregulated genes between Sopt and WT. (E) Significant enrichment COG scatter diagram of differentially downregulated genes between Ropt and WT. (F) Significant enrichment COG scatter diagram of differentially upregulated genes between S and WT. The vertical axis represents the function annotation information, and the horizontal axis represents the rich factor corresponding to the function. The size of Qvalue is represented by the color of the dot. The smaller the Qvalue, the closer the color is to red. The dot size represents the number of differential genes contained in each function.


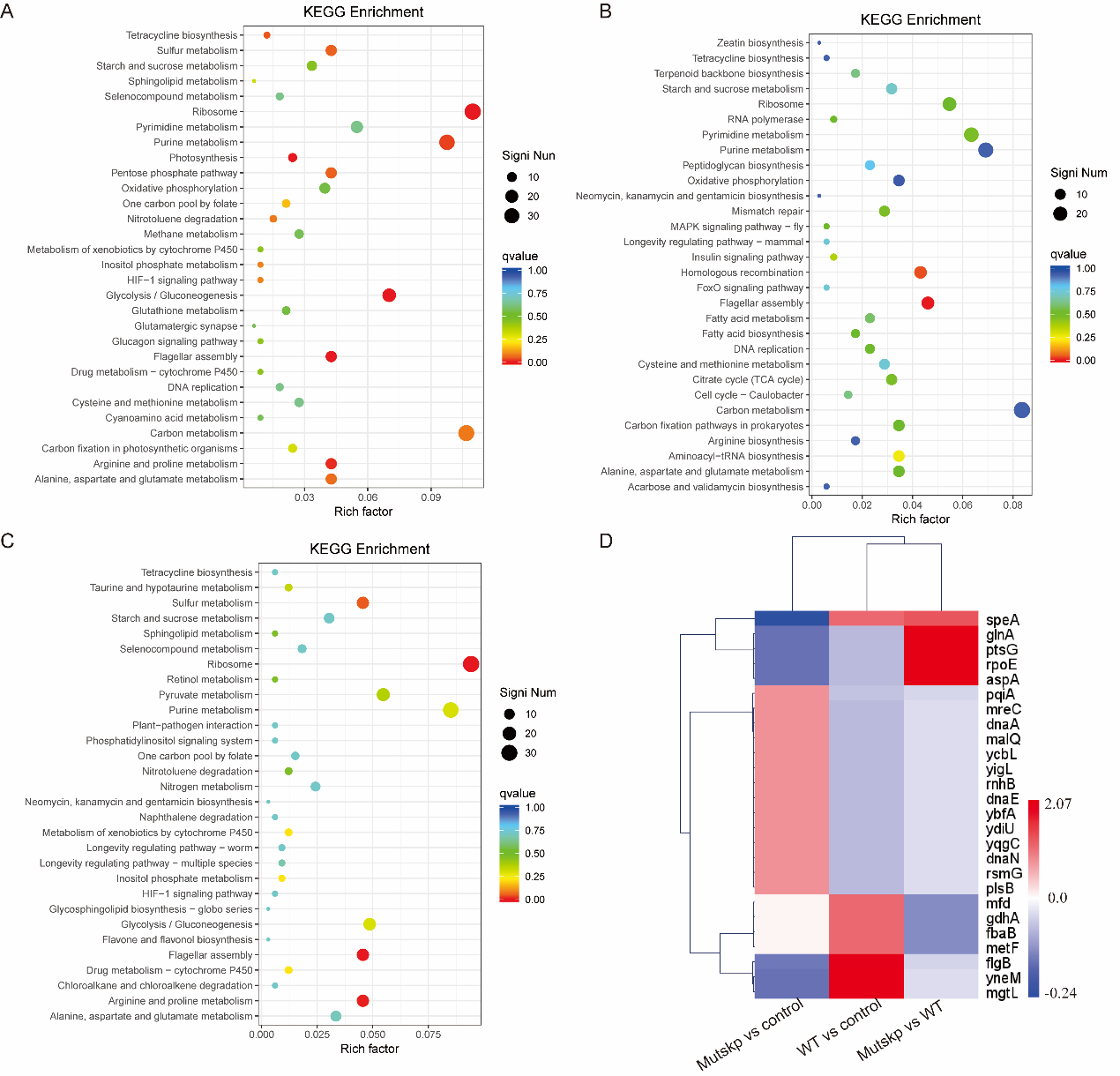


**Fig. S5 Kyoto Encyclopedia of Genes and Genomes (KEGG) analysis of common differential gene. (**A)-(C) Scatter diagram of significant enrichment function of differently expressed genes in the metabolic pathways between Sopt and WT, Sopt and S, Ropt and WT, respectively. The vertical axis represents the function annotation information, and the horizontal axis represents the rich factor corresponding to the function. The size of Qvalue is represented by the color of the dot. The smaller the Qvalue, the closer the color is to red. The dot size represents the number of differential genes contained in each function. (D) Heatmap showing differentially expressed genes (fold change 3, adjusted p-value≤0.05) between *E. coli* expressing LOX, *E. coli* co-expressing LOX with the wild-type skp and *E. coli* co-expressing LOX with mutants of Skp. Rows (genes) were clustered hierarchically.

**Table S1 Plasmids and strains used in this work**

| Plasmid/Strain | Relevant characteristics | Sources or references |
| --- | --- | --- |
| Plasmid |  |  |
| pET Duet-1 | Expression vector, ampicillin resistance | Lab stock |
| pACYC Duet-1 | Expression vector, chloramphenicol resistance | Lab stock |
| pET Duet-1-GFP_1-10_ | pET Duet-1 carring the 1-10^th^ sheet fragment *gfp* gene | This work |
| pET Duet-1-GFP_1-9_ | pET Duet-1 carring the 1-9^th^ sheet fragment *gfp* gene | This work |
| pET Duet-1-GFP_10-11_ | pET Duet-1 carring the 10-11^th^ sheet fragment *gfp* gene | This work |
| pET Duet-1-GFP_11_ | pET Duet-1 carring the 11^th^ gragment of *gfp* gene | This work |
| pET Duet-1-GFP_1-10_/GFP_11_ | pET Duet-1 carring the 1-10^th^ sheet fragment *gfp* gene in the 1^st^ ORF, and carring the 11^th^ gragment of *gfp* gene in the 2^nd^ ORF. | This work |
| pET Duet-1-GFP_1-9_/GFP_10_-LOX-GFP_11_ | pET Duet-1 carring the 1-9^th^ sheet fragment *gfp* gene in the 1^st^ ORF, and carring the 10^th^ and 11^th^ gragment of *gfp* gene at the C- and N-terminus of *lox* gene in the 2^nd^ ORF. | This work |
| pET Duet-1-GFP_1-10_/LOX- GFP_11_ | pET Duet-1 carring the 1-10^th^ sheet fragment *gfp* gene in the 1^st^ ORF, and carring the 11^th^ gragment of *gfp* gene at the C- terminus of *lox* gene in the 2^nd^ ORF. | This work |
| pET Duet-1-GFP_1-10_/GFP_11_-LOX | pET Duet-1 carring the 1-10^th^ sheet fragment *gfp* gene in the 1^st^ ORF, and carring the 11^th^ gragment of *gfp* gene at the N- terminus of *lox* gene in the 2^nd^ ORF. | This work |
| pET Duet-1-GFP_1-10_/ LOX | pET Duet-1 carring the 1-10^th^ sheet fragment *gfp* gene in the 1^st^ ORF, and carring the *lox* gene in the 2^nd^ ORF. | This work |
| pET Duet-1-LOX | pET Duet-1 carring *lox* gene | This work |
| pET Duet-1-GFP_11_-LOX | pET Duet-1 carring the 11^th^ β sheet fragment of *gfp gene* and *lox gene* | This work |
| pACYC-RpoB | pACYC Duet-1 carring *rpoB* gene | This work |
| pACYC-RpoC | pACYC Duet-1 carring *rpoC* gene | This work |
| pACYC-RpoD | pACYC carring *rpoD* gene | This work |
| pACYC-RpoH | pACYC Duet-1 carring *rpoH* gene | This work |
| pACYC-TF | pACYC Duet-1 carring *tf* gene | This work |
| pACYC-DnaK | pACYC Duet-1 carring *dnak* gene | This work |
| pACYC-DnaJ | pACYC Duet-1 carring *dnaj* gene | This work |
| pACYC-GrpE | pACYC Duet-1 carring *grpe* gene | This work |
| pACYC-GroES | pACYC Duet-1 carring *groes* gene | This work |
| pACYC-GroEL | pACYC Duet-1 carring *groel* gene | This work |
| pACYC-IbpA | pACYC Duet-1 carring *ibpa* gene | This work |
| pACYC-IbpB | pACYC Duet-1 carring *ibpb* gene | This work |
| pACYC-ClpA | pACYC Duet-1 carring *clap* gene | This work |
| pACYC-ClpB | pACYC Duet-1 carring *clpb gene* | This work |
| pACYC-HtpG | pACYC Duet-1 carring *htpg* gene | This work |
| Strain |  |  |
| *E. coli* JM109 | The cloning host for plasmid construction | Lab stock |
| *E. coli* BL21(DE3) | The expression host for protein expression | Lab stock |
| *E. coli* ABS/LOX | *E. coli* BL21(DE3) carring pET22b(+)-parABS/LOX plasmid | Pang et al. (2020) |
| *E. coli* Eopt | *E. coli* BL21(DE3) carring pET Duet-GFP_1-10_/GFP_11_-LOX and molecular chaperone plasmid with optimal mutant of GroES | This work |
| *E. coli* Sopt | *E. coli* BL21(DE3) carring pET Duet-GFP_1-10_/GFP_11_-LOX and molecular chaperone plasmid with optimal mutant of Skp | This work |
| *E. coli* Ropt | *E. coli* BL21(DE3) carring pET Duet-GFP_1-10_/GFP_11_-LOX and molecular chaperone plasmid with optimal mutant of RpoH | This work |
| *E. coli* REopt | *E. coli* BL21(DE3) carring pET Duet-GFP_1-10_/GFP_11_-LOX and molecular chaperone plasmid with optimal mutant of GroES and RpoH | This work |

**Table S2 Oligonucleotides used in this study**

| Primer | Sequence |
| --- | --- |
| ibpA-Duet-F | CTTTAATAAGGAGATATAATGCGTAACTTTGATTTATCCCCGC |
| ibpA-Duet-R | GATTACTTTCTGTTCGATTAGTTGATTTCGATACGGCGCG |
| ibpB-Duet-F | TAATAAGGAGATATAATGCGTAACTTCGATTTATCCCCAC |
| ibpB-Duet-R | TACTTTCTGTTCGATTAGCTATTTAACGCGGGACGTTCG |
| DnaK-Duet-F | TAATAAGGAGATATAATGGGTAAAATAATTGGTATCGACCTG |
| DnaK-Duet-R | GATTACTTTCTGTTCGATTATTTTTTGTCTTTGACTTCTTCAAATTCAGCG |
| GroEL-Duet-R | GATTACTTTCTGTTCGATTACATCATGCCGCCCATGC |
| GroEL-Duet-F | TAATAAGGAGATATAATGGCAGCTAAAGACGTAAAATTCG |
| GroES-Duet-F | CTTTAATAAGGAGATATAATGAATATTCGTCCATTGCATGATCGC |
| GroES-Duet-R | GATTACTTTCTGTTCGATTACGCTTCAACAATTGCCAGAATGTCG |
| GrpE-Duet-F | TAATAAGGAGATATAATGAGTAGTAAAGAACAGAAAACGCCTG |
| GrpE-Duet-R | GATTACTTTCTGTTCGATTACGCTTTTGCTTTCGCTACAGTTAC |
| p1-22-F | GTGCGGCCGCAAGCTTTCAGATATTGGTGCTCGCCGGGATAC |
| p1-22-R | GAAGGAGATATACATATGCATCATCATCATCATCATGCAGAAGC |
| ClpP-Duet-R | GATTACTTTCTGTTCGATCAATTACGATGGGTCAGAATCGAATCG |
| ClpS-Duet-R | CGATTACTTTCTGTTCGATCAGGCTTTTTCTAGCGTACACAGC |
| ClpA-F | CTTTAATAAGGAGATATAATGCTCAATCAAGAACTGGAACTCAG |
| ClpA-R | CGATTACTTTCTGTTCGATTAATGCGCTGCTTCCGCCTTG |
| RpoE-F | GAATAAGGTATGAGCGAGCAGTTAACGGAC |
| RpoE-R | GTCCGTTAACTGCTCGCTCATACCTTATTC |
| RpoS-F | GTATGAGTCAGAATACGCTGAAAGTTCATG |
| RpoS-R | CATGAACTTTCAGCGTATTCTGACTCATAC |
| RpoN-F | GTATGAAGCAAGGTTTGCAACTCAGGC |
| RpoN-R | GCCTGAGTTGCAAACCTTGCTTCATAC |
| clpA-F | CAATTGGAGGAATAAGGTATGCTCAATCAAGAACTGGAACTCAG |
| clpA-R | GCGGCCGTGTACAATACGATTAATGCGCTGCTTCCGCCTTG |
| fecl-R | CGGCCGTGTACAATACGATCATAACCCATACTCCAGACGGAACAGCAG |
| fecl-F | CAATTGGAGGAATAAGGTATGTCTGACCGCGCCACTACCAC |
| TF-F | GTATGCAAGTTTCAGTTGAAACCACTCAAGGC |
| TF-R | GCCTTGAGTGGTTTCAACTGAAACTTGCATAC |
| skp-F | CAATTGGAGGAATAAGGTGTGAAAAAGTGGTTATTAGCTGCAGGTC |
| skp-R | GCGGCCGTGTACAATACGATTATTTAACCTGTTTCAGTACGTCGGCAG |
| htpG-F | ACAATTGGAGGAATAAGGTATGAAAGGACAAGAAACTCGTGGTTTTCAGTC |
| htpG-R | TACGATTACTTTCTGTTCGATCAGGAAACCAGCAGCTGGTTC |
| pACYC-R | CATTATATCTCCTTATTAAAGTTAAACAAAATTATTTCTACAG |
| pACYC-F | CACCGCTGAGCAATAACTAGC |
| pACYC-2-R | CATATGTATATCTCCTTCTTATACTTAACTAATATAC |
| pACYC-2-F | CTCGAGTCTGGTAAAGAAACCG |
| pET-1-R | CCATGGTATATCTCCTTCTTAAAGTTAAACAAAATTATTTCTAGAG |
| pET-1-F | AAGCTTGCGGCCGCATAATG |
| TF-ssar-F | CAATTGGAGGAATAAGGTATGCAAGTTTCAGTTGAAACCACTCAAGGC |
| ssar-tk-zt-F | TCGTATTGTACACGGCCGCATAATCG |
| TF-ssar-R | GCCGTGTACAATACGATTACTTTCTGTTCGAACCCGCGGAC |
| ssra-F1 | CATTAATTGCGTTGCGCATTGGCTATCACATCCGACACAAATGTTG |
| ssra-R1 | CACCTTTACGGCCCATACCTTATTCCTCCAATTGTTTAAGACTGCG |
| pet-ssra-F1 | ATGGGCCGTAAAGGTGAAGAACTG |
| pet-ssra-R1 | GCGCAACGCAATTAATGTAAGTTAGCTC |
| ssra-F2 | CGGCCGCATAATCGAAATATTGGCTATCACATCCGACACAAATGTTG |
| pet-ssra-R2 | ATTTCGATTATGCGGCCGTGTAC |
| pet-ssra-F2 | ATGACTTCTGAAAAACGTGATCACATGGTTC |
| ssra-R2 | CGTTTTTCAGAAGTcatACCTTATTCCTCCAATTGTTTAAGACTGCG |
| ssra-tk-F | ATTGGCTATCACATCCGACACAAATG |
| ssra-tk-R | ACCTTATTCCTCCAATTGTTTAAGACTGCG |
| pacyc-ssar-R | CGGATGTGATAGCCAATGCGCAACGCAATTAATGTAAGTTAGC |
| pacya-clpA-F | CAATTGGAGGAATAAGGTATGCTCAATCAAGAACTGGAACTCAG |
| pacya-clpB-F | CAATTGGAGGAATAAGGTATGCGTCTGGATCGTCTTACTAATAAATTC |
| pacya-dnaJ-F | CAATTGGAGGAATAAGGTATGGCTAAGCAAGATTATTACGAGATTTTAGGC |
| pacya-dnaK-F | CAATTGGAGGAATAAGGTATGGGTAAAATAATTGGTATCGACCTG |
| pacya-GroEL-F | CAATTGGAGGAATAAGGTATGGCAGCTAAAGACGTAAAATTCG |
| pacya-GroES-F | CAATTGGAGGAATAAGGTATGAATATTCGTCCATTGCATGATCGC |
| pacya-GrpE-F | CAATTGGAGGAATAAGGTATGAGTAGTAAAGAACAGAAAACGCCTG |
| pacya-htpG-F | CAATTGGAGGAATAAGGTATGAAAGGACAAGAAACTCGTGGTTTTC |
| pacya-ibpA-F | CAATTGGAGGAATAAGGTATGCGTAACTTTGATTTATCCCCGC |
| pacya-ibpB-F | CAATTGGAGGAATAAGGTATGCGTAACTTCGATTTATCCCCAC |
| pacya-TF-F | CAATTGGAGGAATAAGGTATGCAAGTTTCAGTTGAAACCACTCAAG |
| eGroES-F | AACAATTGGAGGAATAAGGTATGAATATTCGTCCATTGCATGATCGC |
| eGroES-R | TACGATTACTTTCTGTTCGATTACGCTTCAACAATTGCCAGAATGTC |
| eskp-F | CAATTGGAGGAATAAGGTGTGAAAAAGTGGTTATTAGCTGCAGGTC |
| eskp-R | GCGGCCGTGTACAATACGATTATTTAACCTGTTTCAGTACGTCGGCAG |
| eRpoH-F | CAATTGGAGGAATAAGGTATGACTGACAAAATGCAAAGTTTAGCTTTAG |
| eRpoH-R | CTTTCTGTTCGACTTAAGCATTTTACGCTTCTATAGCAGCACGC |
| pACYC-F | TCGAACAGAAAGTAATCGTATTGTACACGGC |
| pACYC-R | ACCTTATTCCTCCAATTGTTTAAGACTGCG |

**Table S3 Mutant residue analysis of GroES, Skp and RpoH directed evolution**

| Evolved variant | Mutation |
| --- | --- |
| GroES-1 | G24C, N51D |
| GroES-2 | G24C |
| GroES-3 | E82D |
| GroES-4 | K15I, G24C |
| GroES-5 | E50K |
| GroES-6 | K13T, I63T |
| GroES-7 | K13T, I63T, E82D |
| GroES-8 | R9S |
| GroES-9 | N51D |
| GroES-10 | K15R |
| Skp-1 | A98S |
| Skp-2 | R59H, A98V |
| Skp-3 | Q34L |
| Skp-4 | A98V |
| Skp-5 | Q103I |
| Skp-6 | R59H |
| Skp-7 | M60K, K69N |
| Skp-8 | R59H, K69N |
| Skp-11 | L12V, M60L |
| Skp-15 | N110K |
| RpoH-1 | G72C |
| RpoH-2 | M156I |
| RpoH-3 | R100L |
| RpoH-4 | L139M |
| RpoH-6 | M95I, E271G |
| RpoH-7 | Q230H |
| RpoH-9 | G85A |
| RpoH-10 | P95Q |
| RpoH-11 | G85C |
| RpoH-12 | R122G, Q144L |
| RpoH-17 | R59L |
| RpoH-18 | L146P |
| RpoH-19 | W108L |
| RpoH-20 | R66S |

**Table S3 Analysis of LOX expression level by Image Lab**

| Sample | LOX band area | Increase multiple |
| --- | --- | --- |
| LOX | 1.9×10^6^ | 1.0 |
| LOX-GroES^opt^ | 3.4×10^6^ | 1.1 |
| LOX-Skp^opt^ | 2.0×10^6^ | 1.2 |
| LOX-RpoH^opt^ | 2.2×10^6^ | 1.6 |
| LOX-GroEL-GroES^opt^ | 3.1×10^6^ | 1.8 |
| LOX-RpoH^opt^-ES^opt^ | 3.6×10^6^ | 1.9 |
| LOX-RpoH^opt^-Skp^opt^ | 3.4×10^6^ | 1.8 |
| LOX-RpoH^opt^-EL-ES^opt^ | 3.5×10^6^ | 1.8 |

File S1 Protein sequence of LOX

1 MHHHHHHAEA EAKAKAEAEA KAKWISPTPP TTPTPPTTPT PTPAMDNDSI FFSPLKYLGA

61 EQQRSIDASR SLLDNLIPPS LPQYDNLAGK LARRAVLTSK KLVYVWTENF GNVKGVPMAR

121 SVPLGELPNV DWLLKTAGVI VELIVNFVAS LPASAAAQFE RIATGLSGDL EAARQVHEAL

181 LEEAKNDPAA AGSLLLRFTE LQTRVIAILT RVGLLVDDIL KSASNLVTQR GQGDGLNRFR

241 AVFGTLRLPE VADSFRDDEA FAYWRVAGPN PLLIRRVDAL PANFPLGEEQ FRRVMGADDS

301 LLEAAASRRL YLLDYAELGK LAPSGAVDKL LTGTGFAYAP IALFALGKDR ARLLPVAIQC

361 GQDPATHPMF VRPAESESDL YWGWQMAKTV VQVAEENYHE MFVHLAQTHL VSEAFCLATQ

421 RTLAPSHPLH VLLAPHFEGT LFINEGAARI LLPSAGFIDV MFAAPIQDTQ ATAGGNRLGF

481 DFYRGMLPES LKARNVDDPL ALPDYPYRDD GLLVWNAIRQ WAADYVAVYY ASDGDVTADV

541 ELAAWVGEVI GSGKVAGFRP ITGRSQLVEV LTMVIFTASA QHAAVNFPQP SMMTYAPAIC

601 AMSAAPAPDS PSGKSEADWL KMMPPTLVAL EKVNIYHLLG SVYHGRLGDY RQTGFPYAPV

661 FSDRRVTASG GPLERFQARL KEVEATIRTR NQARRRPYEY LLPSRIPAST NI*
